# A narrative review on obstructive sleep apnoea syndrome in paediatric population

**DOI:** 10.3389/fneur.2024.1393272

**Published:** 2024-07-05

**Authors:** Benedetta Vaienti, Marco Di Blasio, Luisa Arcidiacono, Antonio Santagostini, Alberto Di Blasio, Marzia Segù

**Affiliations:** ^1^Department of Medicine and Surgery, University Center of Dentistry, University of Parma, Parma, Italy; ^2^Department of Biomedical, Surgical and Dental Sciences, University of Milan, Milan, Italy

**Keywords:** paediatric obstructive sleep apnoea, sleep disordered breathing, children, epidemiology, diagnosis, treatment

## Abstract

Obstructive sleep apnoea syndrome is a respiratory sleep disorder that affects 1–5% of children. It occurs equally in males and females, with higher incidence in school age and adolescence. OSAS may be caused by several factors, but in children, adenotonsillar hypertrophy, obesity, and maxillo-mandibular deficits are the most common. In general, there is a reduction in the diameter of the airway with reduced airflow. This condition worsens during sleep due to the muscular hypotonia, resulting in apnoeas or hypoventilation. While snoring is the primary symptom, OSAS-related manifestations have a wide spectrum. Some of these symptoms relate to the nocturnal phase, including disturbed sleep, frequent changes of position, apnoeas and oral respiration. Other symptoms concern the daytime hours, such as drowsiness, irritability, inattention, difficulties with learning and memorisation, and poor school performance, especially in patient suffering from overlapping syndromes (e.g., Down syndrome). In some cases, the child’s general growth may also be affected. Early diagnosis of this condition is crucial in limiting associated symptoms that can significantly impact a paediatric patient’s quality of life, with the potential for the condition to persist into adulthood. Diagnosis involves evaluating several aspects, beginning with a comprehensive anamnesis that includes specific questionnaires, followed by an objective examination. This is followed by instrumental diagnosis, for which polysomnography is considered the gold standard, assessing several parameters, including the apnoea-hypopnoea index (AHI) and oxygen saturation. However, it is not the sole tool for assessing the characteristics of this condition. Other possibilities, such as night-time video recording, nocturnal oximetry, can be chosen when polysomnography is not available and even tested at home, even though with a lower diagnostic accuracy. The treatment of OSAS varies depending on the cause. In children, the most frequent therapies are adenotonsillectomy or orthodontic therapies, specifically maxillary expansion.

## 1 Introduction

Paediatric Obstructive Sleep Apnoea (OSA) is characterised by intermittent complete or partial obstruction (obstructive apnoea or hypopnoea); prolonged partial obstruction of the upper airway; or both prolonged and intermittent obstructions that disrupt normal ventilation during sleep, normal sleep patterns, or both (1).

Obstructive sleep apnoea is part of a group of sleep disorders with a broad spectrum of clinical presentations (2), including Primary Snoring, Upper Airway Resistance Syndrome (UARS), Obstructive Hypoventilation (OH) and Obstructive Sleep Apnoea Syndrome (OSAS).

Primary snoring occurs when snoring is not accompanied by ventilation abnormalities such as apnoea, hypopnoea, hypoxia or hypercapnia (3). Upper airway resistance syndrome (UARS) is characterised by higher intrathoracic pressure during inspiration without apparent apnoea or hypopnoea, resulting in increased respiratory arousal, sleep fragmentation and daytime sleepiness (4). Obstructive hypoventilation (OH) is common in obese children and is diagnosed by snoring, decreased ventilatory drive with hyperventilation without apparent sleep apnoea and respiratory arousal (5).

In order to synthesise the existing knowledge on the various aspects of this topic, a comprehensive literature search was conducted across a range of scientific databases, including PubMed, Google Scholar, the Cochrane Library and Web of Science. Only articles written in English were considered.

Some of the keywords employed included “paediatric obstructive sleep apnoea,” “sleep disordered breathing children” and a combination of the former with the terms “epidemiology,” “symptoms,” “diagnosis,” and “treatment.”

Ultimately, a total of 79 were selected, spanning the period from 1983 to 2024 and available in full-text format.

## 2 Epidemiology

The prevalence of Sleep-Disordered Breathing (SBD) in paediatric population is high, but varies based on the gravity of the pathology, from 8–27% for primary snoring, to 1–4 or 5% for OSAS (6, 7).

Prevalence also changes with other concomitant conditions, such as in Down Syndrome.

Lee et al. (8) analysed in a systematic review of 1,200 paediatric patients with Down Syndrome prevalence of OSAS based on an AHI > 1, 1.5, 2, 5, and 10 events/h is 69, 76, 75, 50, and 34%, respectively. In Down Syndrome there are numerous predisposing factors for OSAS, such as macroglossia, maxillary hypoplasia, obesity, and deficit in muscle tone (8–10).

Ethnic differences may also affect prevalence. Some studies report differences in prevalence and severity of symptoms in African-American individuals compared with the Caucasian population (11, 12). This appears to be related both to a different unfavourable craniofacial conformation, but also to a different pattern of neuromotor control of the pharyngeal region (13).

Prevalence data do not change significantly in Asia compared with the European Caucasian population (14, 15).

Unlike adults, there is no difference between gender in SBD distribution (16).

Sleep-Disordered Breathing in the paediatric population seems to have two main peaks; the first in children aged 2–8 years, in relation to the increase in size of adenoids and tonsils (17).

The second age group concerns adolescents, when substantial weight gain occurs (17).

The great variability of these data is mainly due to the diversity of the analysis methodology used to identify the presence of OSAS and SBD in general (7, 18).

A further bias in estimating the prevalence of these diseases may lie in the fact that the obese population among children is increasing significantly and this is a risk factor for OSAS and SBD in general (2, 19). In addition, the orthodontic population has a higher prevalence of SDB, including OSAS (7). This is explained by the fact that patients seeking orthodontic treatment frequently have certain craniofacial features that increase the risk of these conditions as they are associated with upper airway narrowing (7). These include retrognathia, micrognathia, transversal maxillary defect, midface hypoplasia, macroglossia, increased facial height (7, 20, 21).

## 3 Aethiology and risk factors

The aetiology of this pathology is variable, as airway obstruction may be due to several predisposing factors. First of all, possible anatomical causes that lead to a major collapsibility or narrowing of the airway must be considered; in children, one of the most frequent situations is adenotonsillar hypertrophy, especially in pre-school children (2–5 years) (19).

The presence of craniofacial structures that present a narrowing of the upper airway, such as maxillary hypoplasia, micrognathia, retrognathia, macroglossia, should also be considered (19, 20, 22).

The presence of particular syndromes (19, 22) can lead to malformations or malfunctions of certain structures involved in breathing, such as the presence of hypotonia of the pharyngeal musculature, reduction in the diameter of the pharynx and alterations of the mid-facial structure (19, 22, 23). Examples are Down syndrome, Prader-Willi syndrome, Cruzon syndrome and Chiari malformation (19, 22), Pierre-Robin Sequence (2), Moebius Syndrome (24).

There are also a number of pathological conditions related to OSAS.

Obesity is certainly one of these, which is mainly characteristic of school-age children and adolescents (19).

Several mechanisms contribute to a reduced efficiency of the respiratory system, such as an additional accumulation of fat in the tissues surrounding the upper airways, resulting in increased resistance due to a reduction in their diameter (2).

In addition, obese patients have reduced thoracic compliance, partly due to the accumulation of fat in the tissues, partly due to increased respiratory resistance, and partly due to reduced muscular efficiency, particularly of the diaphragm, whose fibres are overstretched by the abdominal fat deposits (25, 26). This condition is greatly exacerbated in the supine position, making breathing even more difficult (25, 26).

This condition delineates a type of patient very close to the adult patient, with characteristics more similar to the latter, such as significant daytime sleepiness and an increased risk of developing metabolic syndromes and cardiovascular problems (19, 22).

Frequent inflammation of the upper airways, in particular rhinitis and asthma (19), can predispose children to respiratory obstruction during sleep, especially when it occurs in the presence of other risk factors among those mentioned above.

Another category of patients includes those with neuromuscular disorders, which directly affect breathing (19); these include spinal muscular atrophy and Duchenne muscular dystrophy (22). Then there are patients with cerebral palsy, where anatomical and functional factors contribute to airway obstruction and breathing problems (22).

Finally, there are some predisposing environmental factors, such as second-hand smoke (27, 28); there seems to be a correlation between OSAS and reduced vitamin D levels (29), particularly in obese patients, probably related to lifestyle and the comorbidities of obesity (29).

Risk factors are summarised in Table 1.

**Table 1:** Risk factors for paediatric OSAS.

Type of risk factors	Risk factors
Upper airways obstruction risk factors	- Adenotonsillar hypertrophy
Structural and morphological risk factors	- Maxillary hypoplasia - Micrognathia - Retrognathia - Macroglossia
Syndromic risk factors - Down syndrome - Prader-Willi syndrome - Cruzon syndrome - Chiari malformation - Pierre-Robin sequence - Moebius syndrome	- Malformations or malfunctions of certain structures involved in breathing - Presence of hypotonia of the pharyngeal musculature - Reduction in the diameter of the pharynx and alterations of the mid-facial structure
Pathological risk factors	- Obesity - Inflammation of the upper ways (rhinithis, asthma)
Neuromuscular disorders risk factors	- Spinal muscular atrophy - Duchenne muscular dystrophy - Cerebral palsy
Environmental risk factors	- Second hand smoke - Reduced vitamin D

## 4 Clinical presentation and symptoms

The symptoms of OSAS in children are different and extremely variable from patient to patient, both in terms of type and severity of the clinical manifestation.

Generally, sleep is disrupted, although with substantial differences compared to adult patients, as a lower frequency of arousal and 80% apnoea in REM phase (19).

During the Rapid Eye Movement (REM) phase, apnoea is more likely to occur in children due to the generalised muscular hypotonia (19). This is associated with reduced activity of the pharyngeal muscles (30) and a decreased response of the ventilatory system to hypoxia and hypercapnia (31).

Children with sleep apnoea tend to change position frequently during sleep, acquiring even bizarre postures (32, 33), such as hyperextension of the neck (19).

Regarding respiratory symptoms, snoring and mouth breathing are the most common in children with OSAS and are associated with a specific respiratory pattern, namely hypoventilation (2, 19).

Unlike adults, who experience cyclic and prolonged interruptions of breathing, children have an increased central respiratory drive during sleep, which results in enhanced reflexes and muscle tone of the upper airways, leading to reduced airway collapsibility (2).

Another significant difference between adult and paediatric OSAS patients is the cardiovascular risk associated with the disease (2, 19). While certain manifestations, such as cor pulmonale and pulmonary hypertension, can also occur in the paediatric population, these complications are not typically found in children, except for those who are obese and have a family history of heart disease (2, 34).

In children with OSAS, neurocognitive symptoms are significant and can vary in severity (16, 19, 22).

The factors that contribute to the differences in the characteristics and extent of these symptoms are not yet fully understood, but it is believed that certain predisposing phenotypes may play a role (22, 23).

Even objective values from polysomnography are not predictive of the severity of these comorbidities (22).

It is important to note that neurocognitive issues in paediatric patients are established on a developing and maturing nervous system, which may have long-term effects (16). Studies have evaluated this aspect (16).

Symptoms include hyperactivity, inattention, learning difficulties, reduced memory efficiency, poor school performance, and irritability (2, 16, 19).

Excessive daytime sleepiness is reported less commonly in children (17, 35), but if so, then more often, adolescents and obese patients (2, 17, 19); this symptom can also be accompanied by morning headache (36).

Finally, diaphoresis (33, 37), somnambulism (19) and enuresis (38, 39) may be present, particularly in school-aged and preschool children.

As reported above, there is no linear relationship between the severity of OSAS and the severity of associated comorbidities (22, 23). Indeed, it appears that genetic factors, in combination with environmental factors, determine different phenotypes and different degrees of susceptibility and severity of OSAS-related pathological outcomes (22).

Some authors (22) emphasise the difference between obese and non-obese children (22). Obesity is associated with a general state of mild inflammation, which seems to be both enhanced by and enhances the inflammatory response in patients with OSAS (22).

A combination of anorexia and reduced oral intake, increased energy expenditure due to increased work of breathing, and fluctuating nocturnal secretion of growth hormone are likely to contribute to the lack of growth (2, 19, 40).

Numerous studies have demonstrated a correlation between growth recovery and increased secretion of insulin-like growth factor-1 (IGF-1) and IGF-binding protein 3 (IGFBP-3) following adenotonsillectomy (2, 41–43).

## 5 Diagnosis

The diagnosis of Obstructive Sleep Apnoea Syndrome (OSAS) in children is based on various pieces of information. However, the certainty of the presence and severity of the condition can only be obtained through overnight polysomnography (2, 19, 44).

The paediatric population includes individuals aged 18 years and below. However, clinicians may consider patients as adults from the age of 13 years (45).

Parents are typically the primary source of information regarding both nocturnal and daytime signs and symptoms, particularly snoring (2, 46).

Validated questionnaires, such as OSA-18 and PSQ-SRBD, can be useful for screening, but they are not sufficient for diagnosis (47).

The OSA-18 questionnaire specifically investigates the quality of life of both the patient and caregivers (48). It is divided into several sections that analyse different aspects, such as signs and symptoms that occur during sleep (referred to as ‘Sleep Disturbance’); child’s physical problems (“Physical Suffering”), emotional and behavioural aspects (“Emotional Distress”), daytime signs and symptoms (“Daytime Problems”), and the impact of the situation on the child’s caregivers, particularly their anxiety levels and how much it interferes with their daily life (“Caregiver Concerns”) (48).

The PSQ-SRBD questionnaire analyses similar aspects, but only in relation to the child (49–51). It mainly focuses on snoring, daytime sleepiness, changes in weight and growth, and finally behavioural problems such as lack of attention or hyperactivity (49–51).

Questionnaires play an important role in the diagnosis of OSAS by providing extremely useful information (such as night noises, sleep quality, neurocognitive and behavioural aspects, quality of life) and attempting to objectify qualitative data. However, they are not sufficient tools for a definite diagnosis of OSAS.

They can be used as a complement to the patient’s assessment or as an initial screening method to pre-select patients for further analysis.

Furthermore, investigating the caregivers’ behaviour towards the patient can provide insight into the approach to treatment and potential compliance of both the caregivers and the child (48–51).

Additionally, questionnaires can be used to compare the patient’s condition before and after treatment, determining any changes in symptoms and quality of life (48–51).

The medical history is followed by an objective examination to assess the presence of craniofacial features such as micrognathia, maxillary hypoplasia, and macroglossia that may contribute to sleep-disordered breathing.

During the clinical examination, the relationships between the tongue, tonsillar pillars, soft palate, and uvula can be assessed (52, 53).

This analysis relates to two classifications, Mallampati’s and Friedman’s, which were created to evaluate the difficulty of endotracheal intubation (52, 54, 55).

Although both variables show a positive correlation with the presence and severity of OSA, Friedman’s classification appears to have a stronger correlation (52). It is important to note that both variables can be useful in complementing clinical assessments (52).

Other important aspects to be clinically evaluated, even if not directly related to dentistry, include obesity, structural anomalies in the upper respiratory tract, chronic inflammation of the upper airways, neuromuscular pathologies, and specific syndromes such as Down syndrome, Cruzon and Prader Willi (45).

Questionnaires and physical examination alone are not sufficient for making a diagnosis (47). It is estimated that hypothetical diagnoses based solely on questionnaires and objective examinations agree with a positive polysomnography for OSA in only 30–50% of children (47).

Overnight, attended, in-laboratory (44) polysomnography (PSG) is the only objective examination that enables a definitive diagnosis and assessment of the severity of obstructive sleep apnoea syndrome (OSAS) (44, 45, 47); this evaluation is now set as the gold standard (45, 47).

Polysomnography is the only certain means of diagnosis, but unfortunately, it has some limitations, mainly related to cost, duration, stress, and anxiety for young patients (44).

However, it should always be considered, especially for patients with a higher degree of risk, such as obese children, syndromic patients, patients with neuromuscular disorders, and patients with craniofacial abnormalities (47).

The AASM (American Academy of Sleep Medicine) guidelines (56), last modified in July 2023, list the parameters for diagnostic evaluation (Table 2).

**Table 2:** AASM Guidelines for OSAS diagnosis in paediatric population. International Classification of Sleep Disorders Third Edition, Text Revision Summary of Diagnostic Criteria Changes SUMMARY OF DIAGNOSTIC CRITERIA CHANGES.

	Criteria A-C must be met
A	The presence of one of the following: Snoring Labored, paradoxical or obstructed breathing during the child’s sleep Sleepiness, hyperactivity, behavioural problems or learning and other cognitive problems
B	Polysomnography demonstraters one of the following: One ore more apnoeas, mixed apnoeas or hypopneas per hours of sleep A pattern of obstructive hypoventilation, defined at least 25% of total sleep time with hypercapnia (PaCO2 > 50mm Hg) in association with one or more of the following Snoring Flattening of the inspiratory nasal pressure waveform Paradoxical thoracoabdominal motion
C	The symptoms are not better explained by another current sleep disorder, medical disorder, medication or substance use

Polysomnography enables the assessment of multiple parameters in a single examination, such as apnoeas (obstructive, central, mixed), oxygen saturation, end-tidal CO2, sleep staging, sleep architecture, body position and movements, electroencephalogram derivations, and electrocardiogram tracing (45).

After assessing the presence of OSAS, the severity of the condition must be determined. The degree of severity is generally classified according to the AHI (apnoea-hypopnoea index), although there are several parameters to consider (45).

OSAS is classified as mild when the Apnea-Hypopnea Index (AHI) is between 1 and 5, moderate when the AHI is between 5 and 10, and severe when the AHI is greater than 10 (45).

Polysomnography cannot be always performed in children. There are intrinsic issues that may not be overcome in the case of a paediatric patient. It is performed in a hospital setting, modifies the normal bed-routine in children and often changes their sleep quality (57). In this situations or when a preliminary assessment is desired, clinicians may consider optional instrumental examinations.

These factors do not guarantee diagnostic accuracy but may alert the clinician to conduct a more thorough diagnosis.

Examples of such examinations include night-time video recording and night-time oximetry, daytime nap PSG and ambulatory PSG (19, 44).

In circumstances where polysomnography or respiratory polygraphy cannot be conducted due to one (or a combination) of the aforementioned reasons, nocturnal oximetry can be utilised as a screening tool in their stead, as it is relatively simple to perform and is available in numerous diagnostic centres.

This analysis can be performed in the hospital or at home, which is an important advantage for paediatric patients, who are thus at ease in their familiar environment.

Several authors (58–60) have demonstrated the effectiveness of nocturnal oximetry in detecting the presence of obstructive sleep apnoea syndrome (OSAS) based on the number of desaturations in a defined time.

Nixon et al. (59) demonstrated the utility of overnight pulse oximetry in the estimation of OSAS severity, the reduction of diagnostic and therapeutic processes for children presenting with more severe disease.

However, as desaturation is not always present in children with OSAS, a failure to detect the condition by nocturnal oximetry cannot definitively rule it out (59–61). Moreover, nocturnal oximetry is a test that provides only quantitative information on blood oxygen level and heart rate. It does not allow desaturations to be associated with respiratory events and does not allow to distinguish respiratory alterations of obstructive origin from those with other pathogenesis (62).

This final consideration also applies to daytime nap PSG (63, 64), which, although it may be quicker and less costly, carries the risk of underestimating the patient’s situation. This is also due to the fact that the REM phase of sleep can rarely be reached in this type of analysis (63).

Home testing has been proposed to ease the diagnostic process, using portable devices more or less sophisticated, like video recording, devices to record a single channel, such as oximetry, or four channels, such as heart rate, oxygen saturation, respiratory bands, airflow (hRP), or a comprehensive monitoring (hPSG) (63). However, there is currently insufficient evidence to support the use of home testing in children, unlike in the adult population (63, 65, 66).

Analysis of biomarkers has also been proposed, both as screening and monitoring tests, especially with regard to comorbidities and end-organ outcome (67). The proposed biomarkers can be analysed mainly by plasma, few by urine and/or saliva. These analyses relate to factors associated with inflammatory status, such as the products of oxidative stress (63), and the detection and monitoring of cardiometabolic morbidity (68).

## 6 Therapy

The treatment of OSAS is based on the principle of limiting the obstructive and collapsible airway component. Strategies can vary depending on diagnosis, patient and comorbidities.

The most commonly used treatment for sleep apnoea in children is adenotonsillectomy (AT) surgery, since adenotonsillar hypertrophy is the most common cause of this pathology.

This surgery is considered low-risk (44) and effective in patients with adenotonsillar hypertrophy and no comorbidities, with a good success rate, measured by improvement in AHI and OSAS-related symptoms.

On the other hand, success rates have been observed to decrease in the presence of comorbidities, particularly in obese patients (45). Persistent OSAS can be as high as 33–76% in this group, compared to non-obese patients where persistent OSAS after surgery is about 15–37% (45). However, it is important to note that these rates may vary between studies.

Other risk factors for AT resistance, in addition to obesity, include age over 7 years, chronic asthma, severe diseases such as neuromuscular disorders (19, 45), pre-operative AHI ≥ 20 (19).

In cases where mild symptoms persist after AT surgery, intranasal corticosteroids may be administered to reduce mucosal swelling and improve airway patency (44).

The use of intranasal corticosteroids may also be considered as a non-surgical treatment option in cases of mild OSA.

In particular, a systematic literature review with meta-analysis revealed the potential of combining mometasone furoate (an intranasal corticosteroid) and montelukast sodium (a leukotriene receptor antagonist) as an effective treatment option (69). Although further studies are required, the combination of drugs was found to be more effective than other pharmacological treatments in terms of reducing the apnoea-hypopnoea index (AHI) and improving arterial oxygen saturation (69). Furthermore, the drugs were found to be safe in terms of adverse effects (69).

There are also other types of upper airway surgery that directly address possible causing conditions such as turbinate hypertrophy, excess tissue in the soft palate, deviated nasal septum, laryngomalacia (45).

Since maxillary hypoplasia and/or mandibular deficits are common in patients with OSAS, orthodontic therapy also plays a key role in the treatment strategy (2, 19, 45).

In cases of transverse maxillary deficits, maxillary expansion can be carried out using a rapid expander if the child’s bone structure is still growing.

Otherwise, a surgically assisted expansion can be performed to detach the midpalatine suture.

Patients treated with maxillary expansion generally experience improvements in OSAS-related symptoms, as well as objective parameters such as AHI and oxygen saturation (70–72).

Maxillary expansion increases the volume of the nasal cavities, thereby reducing resistance at this level and increasing airflow, stimulating nasal breathing, which limits mouth breathing (70–73).

In addition, the increased size of the palate and maxilla allows for better lingual positioning with improved oropharyngeal patency and better lip seal (70–73).

Staying within the field of orthodontic approaches, in cases of mandibular deficit, mandibular advancement through functional or orthopaedic devices can be considered (45, 74, 75). This can improve lingual positioning and increase patency of the oropharynx, while also enhancing the aesthetic profile (76); this kind of approach is a part of conservative treatment, which is typical in paediatric dentistry (77).

Intervening early on the child’s craniofacial structure, trying to improve its growth, can also try to limit any interventions needed in adulthood, which require far more invasive and complex planning and procedures (78).

When OSAS persists after surgery or other first-line treatments, or when surgery is not an eligible option or is not desired by the patient or their parents, CPAP (Continuous Positive Airway Pressure) may be used.

CPAP is a device used to maintain airway patency by providing a continuous flow of air at positive pressure through a dedicated mask, keeping the airways clear during sleep (44).

CPAP is widely considered the most effective treatment for persistent OSAS, with its benefits far outweighing any adverse effects (44).

It can also be particularly useful in treating obese children, in conjunction with weight loss recommendations, which can be beneficial to both the obstructive pathology and the child’s overall health (44, 45).

Moreover, despite the lack of specific studies in the paediatric population, there is the possibility of combining these primary strategies with speech therapy (79, 80). This combination appears to improve the patient’s quality of life and compliance with CPAP (79, 80).

This device has potentially very good efficacy, which is, however, limited by the low adherence to its use. It can be uncomfortable and cumbersome for many children, resulting in general discomfort (44, 45). Additionally, side effects such as dryness of the mucous membranes, nosebleeds, nasal congestion, and skin abrasions may occur (45).

To date, some authors (81) have identified the potential of utilising an alternative therapeutic strategy, namely high flow therapy (HFT), which is based on the insufflating of air into the upper airways through a nasal cannula.

A recent RCT by Fishman et al. (81) demonstrated that HFT could potentially have comparable efficacy to CPAP therapy in the management of OSAS in obese children or patients with complex medical conditions. The primary outcome was reduction in AHI, while sleep quality, arousal index, nadir oxygen saturation and oxygen desaturation index were evaluated as secondary outcomes.

The study demonstrates that high flow therapy represents an effective and more patient-friendly alternative to CPAP, as it is less cumbersome and more comfortable (81).

Nevertheless, there are some limitations to this approach. For instance, there is a lack of evaluation of the management of this therapy in the home environment. Furthermore, the authors themselves emphasise the need for further studies on this subject (81).

## Author contributions

BV: Writing – review & editing, Writing – original draft, Methodology, Data curation, Conceptualization. MB: Writing – review & editing, Writing – original draft, Methodology, Data curation, Conceptualization. LA: Writing – original draft, Investigation, Data curation. AS: Writing – original draft, Supervision, Data curation. AB: Writing – review & editing, Visualization, Validation, Supervision, Data curation. MS: Writing – review & editing, Validation, Supervision, Formal analysis.
